# Mobile Health (mHealth) for Chronic Low Back Pain Assessment: Design, Development, and Usability Evaluation

**DOI:** 10.1002/jsp2.70118

**Published:** 2025-10-01

**Authors:** Zakiy F. Alfikri, Marit E. Johnson, Brad E. Dicianno, Carol M. Greco, Bambang Parmanto, Sara R. Piva, Rachel E. Roos, Andi Saptono, Gwendolyn A. Sowa, Leming Zhou, Kevin M. Bell

**Affiliations:** ^1^ Department of Bioengineering University of Pittsburgh Swanson School of Engineering Pittsburgh Pennsylvania USA; ^2^ Department of Orthopaedic Surgery, School of Medicine University of Pittsburgh Pittsburgh Pennsylvania USA; ^3^ Department of Health Information Management, School of Health & Rehabilitation Sciences University of Pittsburgh Pittsburgh Pennsylvania USA; ^4^ Department of Physical Medicine & Rehabilitation, School of Medicine University of Pittsburgh Pittsburgh Pennsylvania USA; ^5^ Department of Psychiatry, School of Medicine University of Pittsburgh Pittsburgh Pennsylvania USA; ^6^ Department of Physical Therapy, School of Health & Rehabilitation Sciences University of Pittsburgh Pittsburgh Pennsylvania USA; ^7^ Clinical and Translational Science Institute University of Pittsburgh Pittsburgh Pennsylvania USA; ^8^ Bethel Musculoskeletal Research Center, Ferguson Laboratory for Orthopaedic and Spine Research, School of Medicine University of Pittsburgh Pittsburgh Pennsylvania USA; ^9^ University of Pittsburgh, McGowan Institute Pittsburgh Pennsylvania USA; ^10^ Intelligent Systems Program, School of Computing and Information University of Pittsburgh Pittsburgh Pennsylvania USA

**Keywords:** ecological momentary assessment, low back pain, mobile applications, physical therapy, rehabilitation, telemedicine, user‐centered design

## Abstract

**Background:**

Chronic low back pain (cLBP) requires precise phenotyping for tailored treatments. This study introduces a mobile health (mHealth) system for cLBP assessment, aiming to collect extensive biomechanical and behavioral data from in‐clinic and seven‐day at‐home assessments from 1000 individuals with cLBP to accommodate accurate phenotyping.

**Methods:**

Using a user‐centered design approach, an integrated mHealth system was developed, comprising two mobile applications: a clinician‐facing in‐clinic app and a participant‐facing at‐home app. The in‐clinic app aids physical therapists in conducting in‐clinic assessments, while the at‐home app allows cLBP patients to manage and submit responses to ecological momentary assessments (EMA). Usability evaluations were conducted using the mHealth App Usability Questionnaire (MAUQ) and qualitative open‐ended questions asking about ease of use, learnability, overall impression and satisfaction, and reflective questions. Scores from MAUQ were summarized using median and interquartile range (IQR). The usability results were used to iteratively refine the system's design and functionality.

**Results:**

Three physical therapists and 337 out of 522 cLBP patients participated in the usability evaluations. The evaluations demonstrated positive feedback for both apps. For the in‐clinic app, the first iteration median MAUQ score was 6 (IQR 1) and the second iteration median MAUQ score was also 6 (IQR 2). For the at‐home app, the median MAUQ scores were consistently high across five iterations (median score of 7 (IQR 1) for all iterations). These scores indicated good usability, meaning they were easy to use, efficient, and satisfying. Iterative modifications based on the feedback focused on enhancing navigation consistency, responsiveness, and user interface, resulting in overall improved usability.

**Conclusion:**

The in‐clinic app was successfully used by physical therapists for the assessments of 1000 cLBP patients, receiving positive feedback. Similarly, 989 cLBP patients used the at‐home app to complete and submit their EMA, finding it easier to comply with the assessment.

## Graphical abstract

This study introduces a mobile health (mHealth) system for chronic low back pain (cLBP) assessment, involving in‐clinic and at‐home apps to collect comprehensive data from 1000 individuals. Usability evaluations showed positive feedback, with high median scores indicating good usability for both apps, leading to iterative improvements in navigation, responsiveness, and user interface.

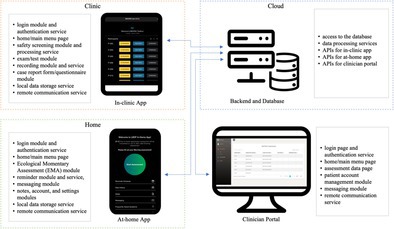

## 1 Introduction

Chronic low back pain (cLBP) is one of the most prevalent conditions in developed countries, including the United States [1, 2, 3, 4]. It is a complex condition influenced by numerous factors, including, but not limited to, biomechanical (e.g., poor posture, movement abnormalities), behavioral (e.g., physical inactivity, pain avoidance behaviors, sleep disturbances), and biological factors (e.g., inflammation, genetic predisposition) [5, 6]. Characterizing low back pain based on these factors into distinct phenotypes is increasingly crucial [7, 8]. Tailoring the treatment approach to the unique characteristics of each cLBP phenotype has the potential to greatly enhance treatment outcomes and reduce costs. Understanding cLBP comprehensively requires careful collection and analysis of various assessment data, including biomechanical, behavioral, and biological information from affected individuals [7].

The use of mobile health (mHealth) technology has been widespread [9, 10, 11, 12, 13, 14, 15], including to facilitate assessment processes [16, 17]. An mHealth system can collect and record a wide range of data types, including, but not limited to, textual data (e.g., pain diaries, patient‐reported notes), multimedia contents (e.g., video recording of the patient performing exercises), and predefined survey responses (e.g., Likert‐scale responses on their daily functioning). This study focused on the development and usability evaluation of two mobile applications for in‐clinic and at‐home assessments within an mHealth system tailored for the comprehensive assessment of cLBP. As part of a broader initiative to collect data from over 1000 cLBP patients [7], this mHealth system was primarily developed to capture biomechanical and behavioral assessment data, which will be discussed in the Method section. These data will contribute to a multifaceted analysis aimed at establishing distinct phenotypes of cLBP.

Existing tools, such as the NIH Toolbox app [18], could assess motor function but did not provide custom tests that may be needed by some cLBP assessments. Additionally, integrating a customized safety screening module and instructional multimedia content into the in‐clinic assessment flow was not feasible with those existing apps. Moreover, given the goal to assess over 1000 cLBP patients, developing our own mHealth system was necessary for better and more streamlined data management and in‐clinic assessment flow. Similarly, developing our own app for at‐home assessment that is integrated with the mHealth system was necessary to easily incorporate a custom EMA module and secure messaging.

The effectiveness of mHealth apps is related to their usability. Adopting a user‐centered approach during the development of mHealth systems is crucial for optimizing their usability [19, 20, 21, 22, 23, 24]. Integrating user feedback and continuous usability evaluations into the iterative development process allows the mHealth system to meet the specific demands of cLBP assessment. The aim of this study is to describe the user‐centered development, including the usability evaluation, of an mHealth system designed for cLBP assessment.

## 2 Methods

This study was approved by the Institutional Review Board of the University of Pittsburgh (STUDY20030093). A total of 522 individuals with cLBP, who actively utilized the at‐home app, were asked to evaluate its usability at the end of their at‐home assessment session. These individuals were a convenience sample of the first 522 out of 1000 cLBP individuals enrolled in the large cLBP phenotyping study [7]. Participants were recruited through routine clinical care, research registries, and public announcements. The inclusion criteria were having cLBP per NIH definition of cLBP [25], being 18 years old or older, and the ability to speak and understand English. The exclusion criteria were participants not listed in our electronic health record system, those in a double‐blind intervention study for LBP, or individuals with conditions that increase risk or lead to non‐compliance.

### 2.1 Requirements and Design Specification

The mHealth system in this study was developed to facilitate a larger study on phenotyping cLBP [7], where in‐clinic and at‐home cLBP physical therapy assessments were conducted. In this mHealth system, an in‐clinic app was designed to handle the in‐clinic assessment, while an at‐home app was designed for the at‐home assessment. These two apps were part of our broader mHealth system, which also included a web portal and a backend database.

To build the in‐clinic app, the outline of the in‐clinic assessment process needs to be thoroughly examined. In the larger study, for the in‐clinic assessment, physical therapists assessed people with cLBP through a specified set of clinical exams (e.g., neurological examination, sacroiliac joint dysfunction test, muscle function tests), functional performance tests (e.g., 4‐m walk, 2‐min walk, 5‐time sit‐to‐stand), and quantitative sensory testing (QST, e.g., pain pressure threshold, pain temporal summation, conditional pain modulation, cold pain tolerance test) [26, 27]. The values of those exams and tests need to be recorded and integrated into the study database. Additionally, many essential resources need to be provided to the physical therapists to help with the in‐clinic assessment, including an exam guide, safety screening rules, assessment checklist, predefined allowable ranges of assessment values, and case report form. All those materials and resources need to be integrated in ways to help the physical therapists conduct the in‐clinic assessments and to streamline and make the in‐clinic assessment flow more efficient.

Based on the process, the ideal scenarios need to be evaluated to inform the development of the in‐clinic app. The in‐clinic protocols and flow should be easily adjusted based on participants' medical and safety screening results while still ensuring that physical therapists follow the protocols. With the pen‐and‐paper method, physical therapists need to adjust and manage protocol changes based on the screening by themselves, which can introduce inefficiency and errors. There are also physical therapists' notes about adverse events and other related notes that are not the main outcomes of the in‐clinic assessments that should be considered. This information should be easily captured and integrated with the overall flow of the in‐clinic assessment.

For the at‐home app development, the at‐home assessment process needs to be reviewed. In the larger study, for the at‐home assessment, patients should be able to report their self‐assessed pain intensity and interference (recorded on a 0–10 Likert scale, where a higher number indicates greater pain intensity/interference), sleep time (sleep and wake‐up time), and activity types (e.g., sport, hobbies, work, home activities) and intensity (very light, light, moderate, moderate to vigorous, and vigorous) three times a day during a 7‐day assessment period in their homes or community settings. They should be able to achieve this with minimal burden to their daily lives. Given that the setting is the patients' homes and their community environments, it is important that the system seamlessly integrates into their routines. The patients should also be able to communicate with the study representatives securely as needed if they have any questions or issues about the at‐home assessment. Additional materials, such as informational content and answers to frequently asked questions, would be good to provide. Their assessment should happen in real time in their natural settings, which can help to minimize recall bias and provide a more accurate picture of the patients' daily lives. Reminders and notifications should also be incorporated to help them comply with the study requirements of submitting their EMA.

### 2.2 User‐Centered System Development

The mHealth system was designed and developed using a user‐centered design approach [20]. The development was conducted in iterations, with usability evaluations in each iteration to assess the system's usability. A prototype was created based on the requirements and needs of the cLBP assessment research project. Feedback from users and usability evaluations was used to make changes and improvements to the app during subsequent development iterations. In this study, the iterations were conducted within 3 months of each other. Although the usability evaluations were conducted within 3 months of each other, any user feedback was addressed as early as possible to support rapid development and gather more feedback in an agile development cycle. This approach allowed the development team to incorporate user feedback at various stages of the process, leading to the creation of a system that is usable, effective, and well‐suited to the needs of the target users.

### 2.3 Usability Evaluation

The assessment process for the at‐home app involved conducting a total of five distinct usability evaluations, each contributing valuable insights and feedback for potential enhancements.

Three physical therapists were assigned to conduct the in‐clinic assessments. They utilized the in‐clinic app during the assessments at the clinic and actively participated in providing feedback and evaluating the in‐clinic app's usability. The evaluation process for the in‐clinic app comprised two formal usability evaluations.

The mHealth App Usability Questionnaire (MAUQ) was employed to evaluate the usability of the two apps. MAUQ is a validated tool [28] specifically designed to evaluate the usability of mobile health applications [29, 30, 31], making it an ideal choice for assessing the user experience and identifying areas for improvement in this study. For the in‐clinic app, the evaluation utilized the MAUQ tailored for standalone mHealth apps used by healthcare providers [32], while the evaluation of the at‐home app utilized the MAUQ designed for standalone mHealth apps used by patients [33]. Open‐ended questionnaires were also used along with MAUQ to assess the usability. The open‐ended questionnaires for both apps had five questions: “How easy do you think it is to perform tasks using this app?”, “How quickly do you think you can perform tasks using this app after the training?”, “How pleasant is it to use the app to perform the tasks?”, “What do you think can be improved from the app?”, and “What is your overall impression in using the app?”. The responses were categorized as either positive or negative feedback by the first author based on the usability components mentioned. Usability issues and suggestions from the responses were also collected and used as guides to further improve the apps in the subsequent iterations of the development process.

## 3 Results

### 3.1 Preliminary System Design

**FIGURE 1:**
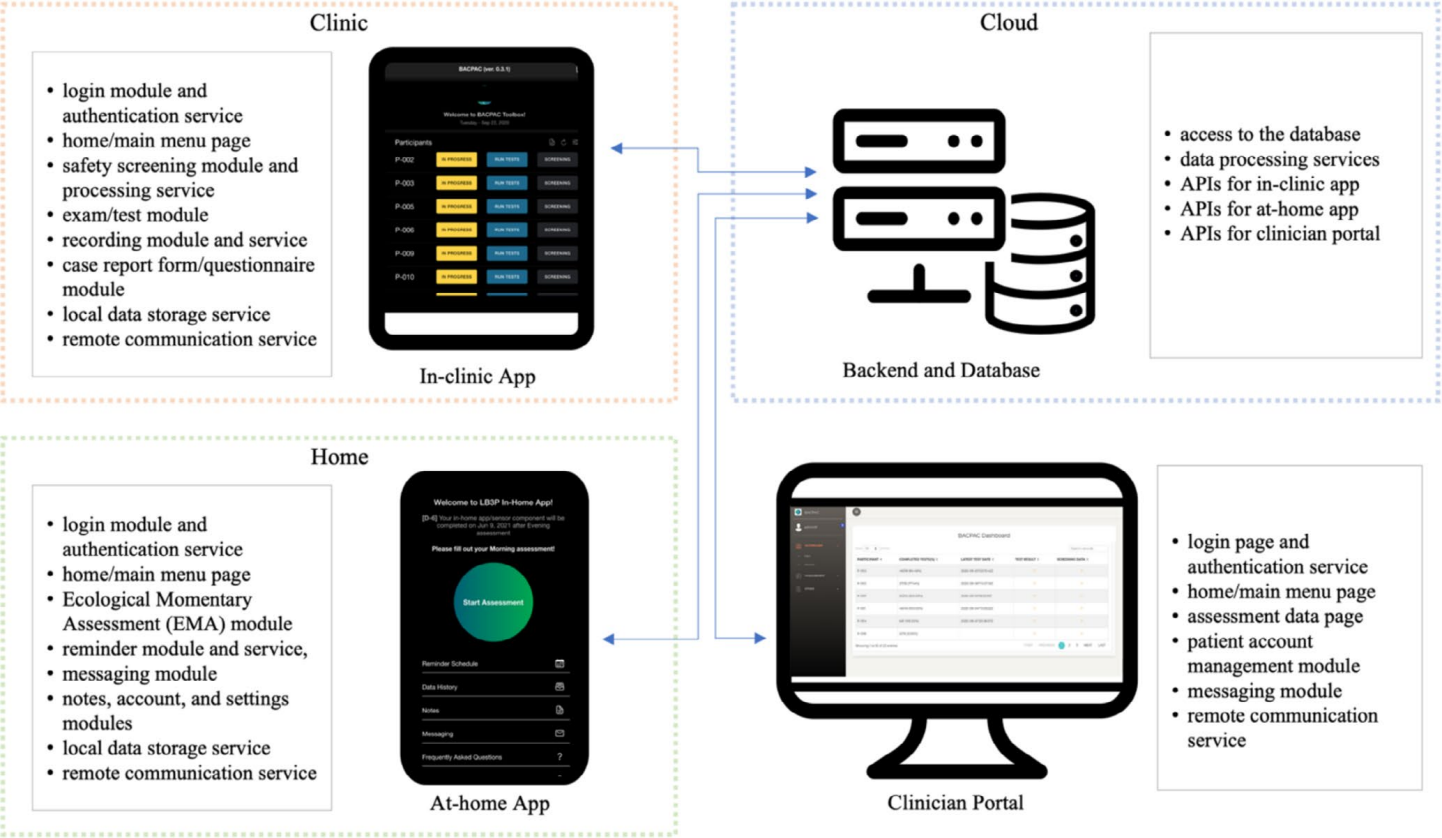
Design of the mHealth system.

The in‐clinic app and at‐home app were designed as part of a broader mHealth system. The details of the relationship and the functionalities of each component in the system are illustrated in Figure 1.

### 3.2 Iterative Development and Usability Evaluation for In‐Clinic App

**FIGURE 2:**
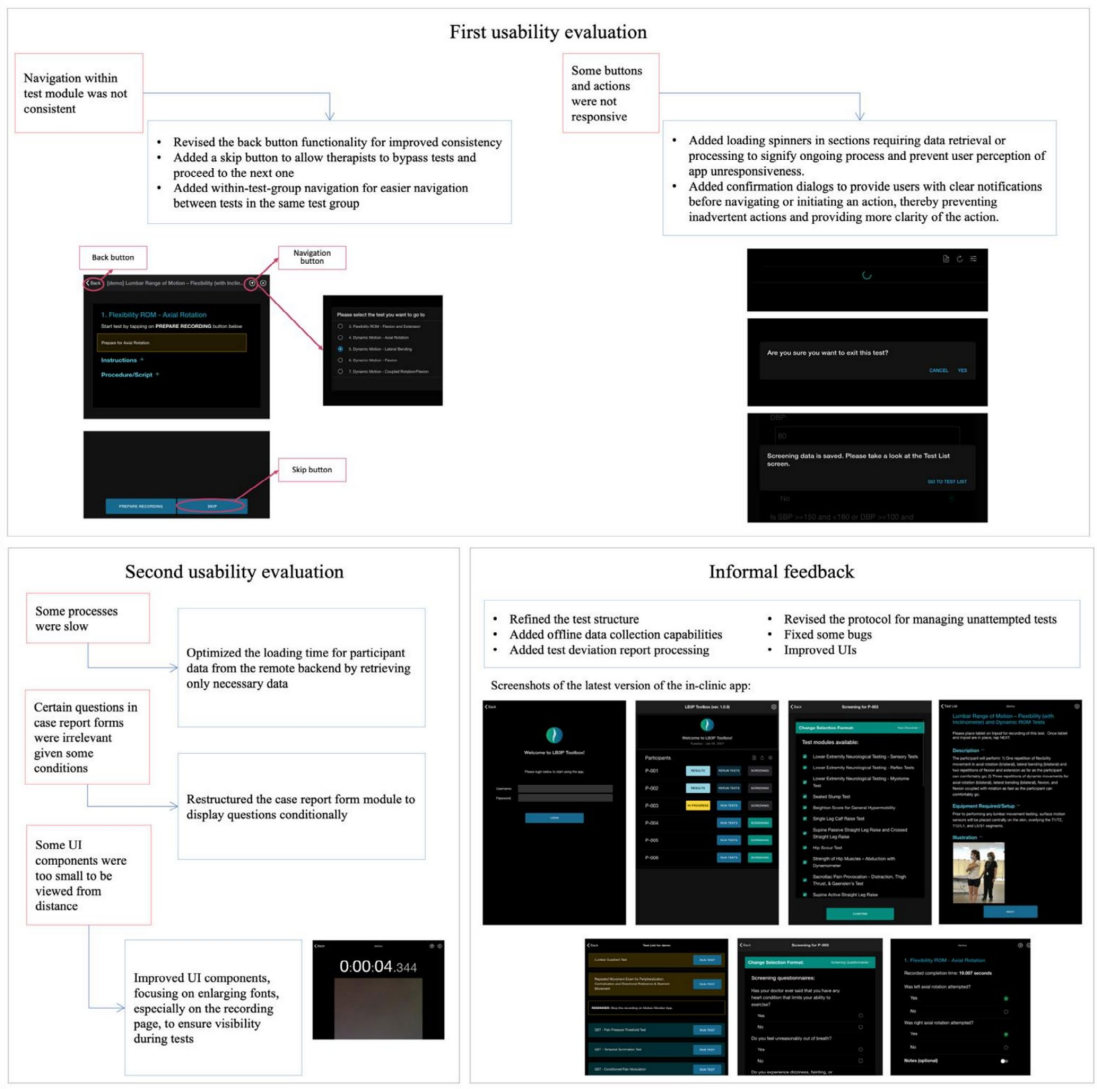
Usability concerns from the physical therapists and in‐clinic app updates for each iteration of the usability evaluation.

The first usability evaluation of the in‐clinic app, completed by three physical therapists, resulted in a median MAUQ score of 6 (IQR 1), indicating positive feedback from the physical therapists. The open‐ended questionnaires also revealed that the overall impression of the physical therapists was positive. They expressed confidence that they would be able to use the app easily if given more time to try it out, and they believed that the app would be helpful for them. However, issues with navigating within the test module and unresponsiveness in certain processes were identified. To address these concerns, significant updates were implemented, primarily focusing on enhancing navigation within the test module and adding UI feedback components, as illustrated in the first box of Figure 2.

The median MAUQ score for the second usability evaluation, completed by one physical therapist, was 6 (IQR 2). This is similar to the first usability evaluation but with a higher upper quartile, reflecting a retained positive impression from the physical therapists and the positive impact of the adjustments made to the app based on their feedback, resulting in a more user‐friendly app. Analysis of the open‐ended questionnaires revealed that the physical therapists had a favorable impression and appreciated the implemented changes. However, they mentioned some areas that required attention. They noted occasional sluggishness in certain app processes and suggested enhancements to the case report form module. They found the task of answering all questions exhausting, particularly when irrelevant questions were included based on the test results. Additionally, they expressed a need for a larger interface to facilitate monitoring the app from a distance while standing next to the participant. Several adjustments to improve the process, restructure the case report form, and enhance the UI were made, as illustrated in the second box of Figure 2.

**FIGURE 3:**
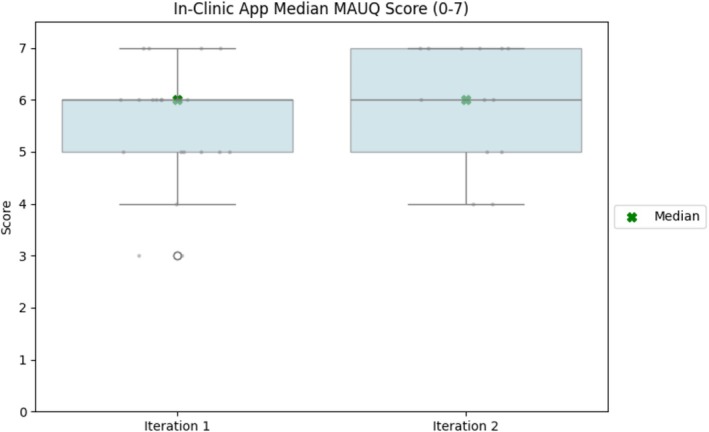
Median MAUQ scores for each iteration of in‐clinic app usability evaluation.

Given the satisfactory usability scores from the initial and subsequent assessments, as illustrated in Figure 3, and since the first author was present with the physical therapists during the in‐clinic assessments to monitor and provide technical assistance, further usability evaluations were not conducted to mitigate potential reviewer bias. However, ongoing data collection and analysis of feedback from the physical therapists continued, leading to the implementation of further changes and enhancements based on their valuable input. Physical therapists provided feedback directly or through the study coordinator, driving a series of updates and improvements, which included refining the test structure, introducing offline data collection capabilities, implementing test deviation report processing, and revising the protocol for managing unattempted tests. Additionally, several bug fixes and UI enhancements were integrated, informed by the feedback provided by the physical therapists.

### 3.3 Iterative Development and Usability Evaluation for At‐Home App

Five usability evaluations were conducted for the at‐home app. The app's usability module was presented to the patients following their 7‐day at‐home assessment session. Patients were encouraged to fill out the usability questionnaire at the end of their assessment, allowing them ample time to experience the app. Although completing the usability questionnaire was optional, 337 out of 522 patients filled out the MAUQ questionnaire. They are grouped into five evaluation iterations based on the period they used the app. Each group had different individuals.

**FIGURE 4:**
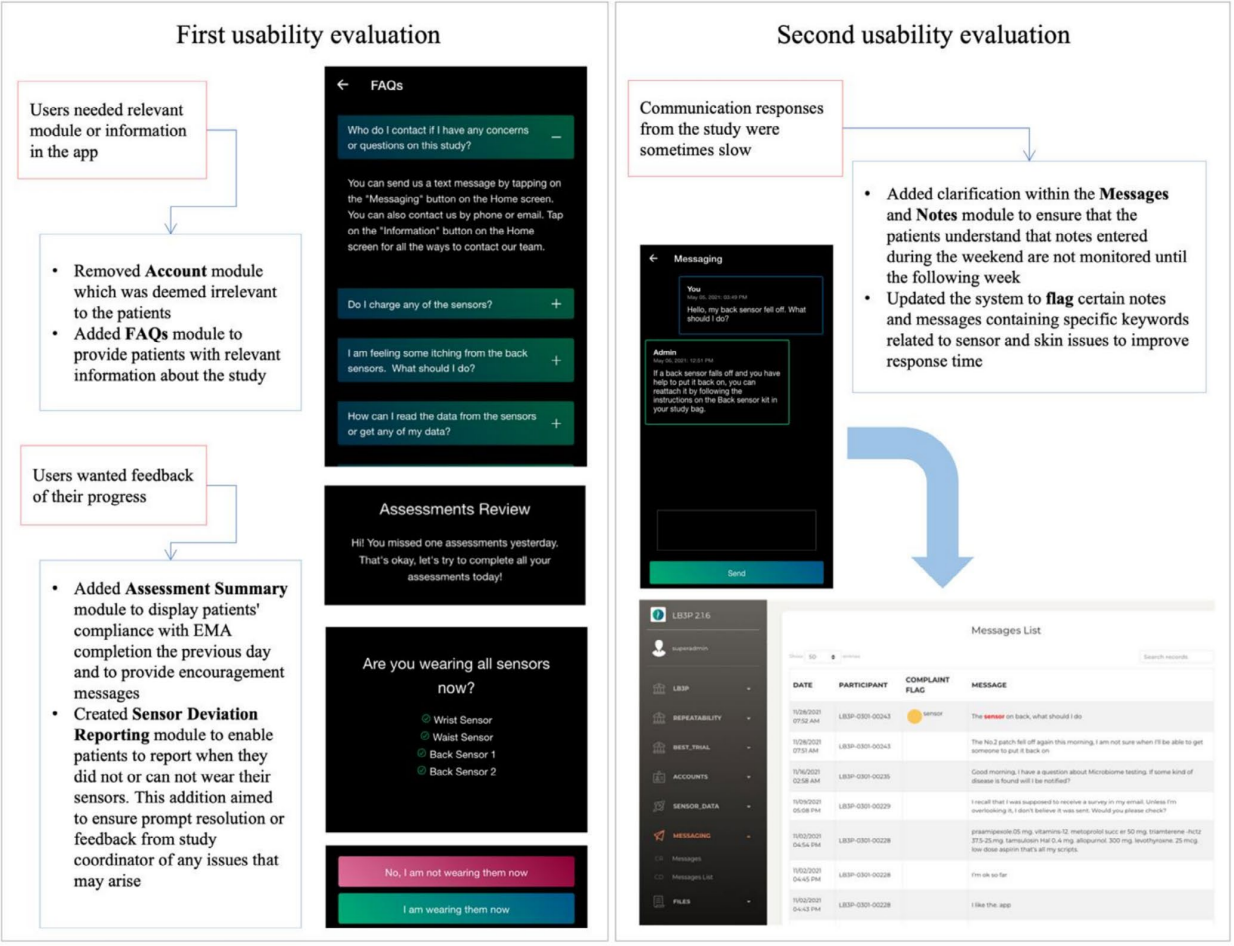
Usability concerns and at‐home app updates for each iteration of the usability evaluation.

In the initial usability evaluation, 68 out of 193 eligible 193 patients submitted their questionnaire responses, resulting in a median MAUQ usability score of 7 (IQR 1). These findings indicate that the initial functional version of the at‐home app exhibited good usability. Analysis of the open‐ended questionnaires revealed that overall, patients had a positive impression of the app. Some patients proposed improvements, including the option to fill out the EMA retrospectively. However, this feedback was not incorporated, as the EMA's purpose is to assess patients in real time rather than retrospectively. Subsequent adjustments were made to the app based on the feedback. Feedback on requesting more relevant modules and getting feedback on the study progress and compliance was addressed, as illustrated in the first box of Figure 4.

In the second usability evaluation, conducted 3 months after the initial at‐home app usability assessment, 62 out of 74 eligible patients completed the usability questionnaire. The resulting median score was 7 (IQR 1), showing minimal change from the first assessment. Based on feedback from patients and study coordinators, several modifications were made to the app. Patients' feedback on communication was addressed by updating the messaging system and protocols as illustrated in the second box of Figure 4.

**FIGURE 5:**
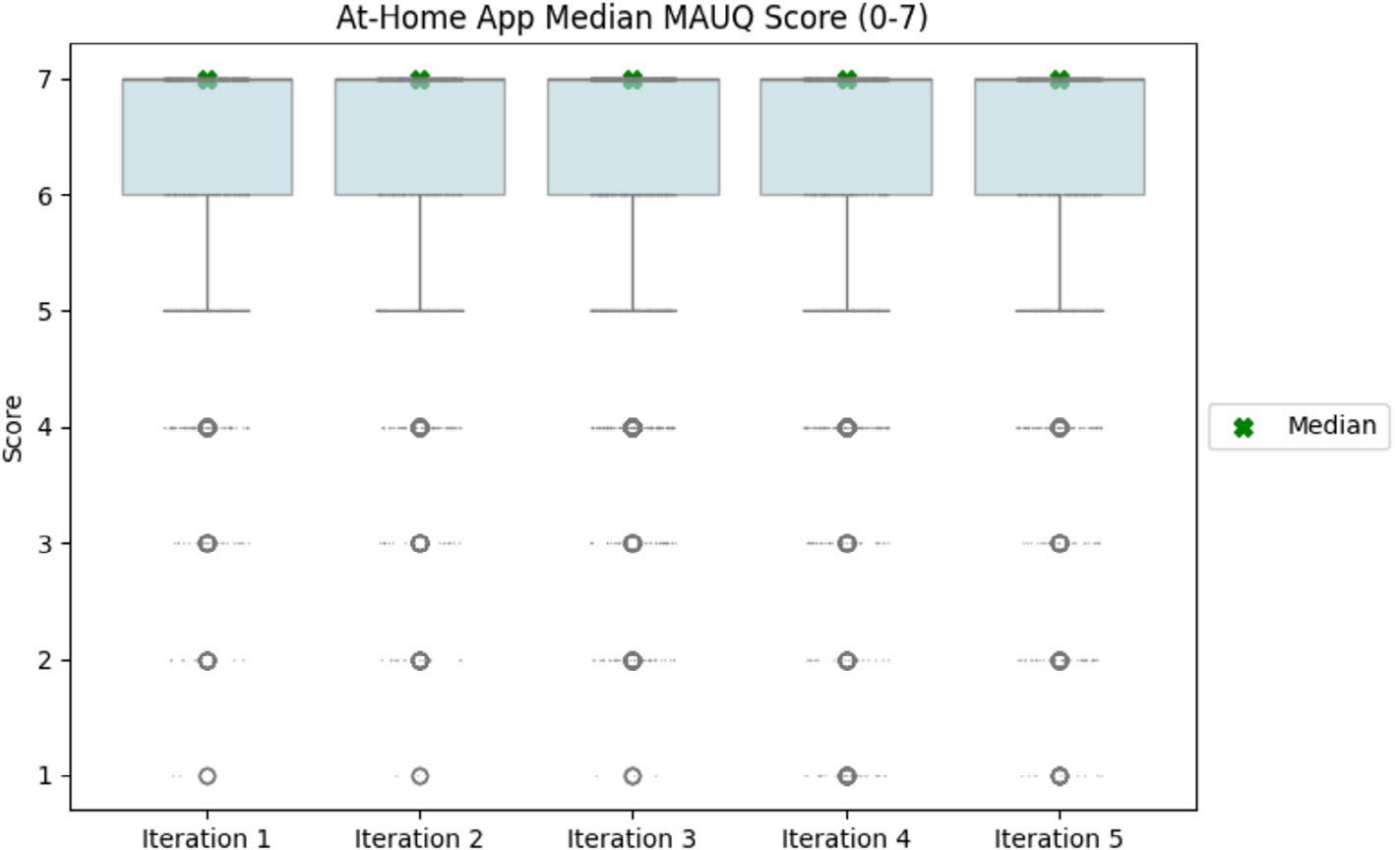
MAUQ scores for each iteration of at‐home app usability evaluation.

Subsequent usability evaluations, 3 months apart, were conducted to ensure ongoing app performance. The third evaluation, with 54 out of 65 patients, resulted in a median score of 7 (IQR 1). The fourth evaluation, with 80 out of 98 patients, resulted in a median score of 7 (IQR 1). The fifth evaluation, with 73 out of 92 patients, resulted in a median score of 7 (IQR 1). As the evaluations progressed, the scores demonstrated a consistent plateau, showing a consistent usability performance, as can be seen in Figure 5. Throughout these evaluations, the app continued to effectively serve its intended purposes for patients, study coordinators, and researchers involved in the data collection process within the at‐home app.

## 4 Discussion

### 4.1 Principal Findings

The in‐clinic app has demonstrated to be a valuable asset for physical therapists, significantly enhancing the efficiency and effectiveness of in‐clinic assessments. As of the time of publication, the app has facilitated the assessment of over 1000 patients, demonstrating its potential for widespread adoption and utility. An important opportunity identified is the potential for the in‐clinic app to be utilized in other similar studies, expanding its applicability and impact in various research contexts. The at‐home app effectively facilitated patient engagement, enabling the seamless completion and submission of EMA. The at‐home app has shown promise in improving patient engagement, ensuring that they adhere to study protocols more consistently. Out of the 989 participants who used the at‐home app, they completed an average of 81.8% ± 23.0% of their assessments, which equates to approximately 17 out of 21 assessments.

This study confirmed that the user‐centered development approach resulted in apps with good usability. Both the in‐clinic and at‐home apps were well received by the users, highlighting the effectiveness of incorporating user feedback throughout the development process. This approach has led to the creation of tools that are not only functional but also user friendly, meeting the needs of both physical therapists and patients. These findings align with existing literature, which emphasizes the importance of usability and user‐centered design in the development of mHealth apps [34, 35, 36]. The positive feedback and high usability scores from our study support this notion, demonstrating that involving end users in the development process can lead to more effective and widely accepted mHealth apps.

Compared to other cLBP assessment studies [37, 38, 39], our study stands out due to the utilization of mobile apps and the ability to conduct both in‐clinic and at‐home cLBP assessments. By using both apps, a wide range of data from these assessments can be easily collected and automatically integrated into one system. This integration makes both data collection and analysis more structured and seamless.

This study has identified several promising opportunities for further development and expansion of the mHealth system. An area of potential growth lies within the in‐clinic app, which has already demonstrated its adaptability and configurability. In addition to the improvements made to address app usability issues, in an ancillary study, changes were also made to expand the app's capabilities for use in other similar studies [40]. These modifications include the incorporation of diverse participant types, allowing for a different range of tests, and the integration of configurable access for different studies within the app.

Additionally, the at‐home app presents another avenue for development, as its current success in aiding patients' assessments suggests the potential for expanding its functionality to include treatment plans. The extensive and rich assessment data collected in this study offer the prospect of developing a machine learning component, facilitating the creation of personalized and adaptive treatments for cLBP. Leveraging this machine learning component, the at‐home app can be further enhanced to incorporate a personalized and adaptive treatment module, thus advancing the system's capabilities from assessment to the provision and delivery of effective treatments for patients with cLBP.

Integrating the apps with motion‐tracking wearables presents a significant opportunity to enhance the comprehensiveness of cLBP assessments. These technologies can improve both the overall system and the assessment process. Several motion‐tracking wearables show great promise in measuring various cLBP‐related metrics [41, 42, 43].

### 4.2 Limitations

Despite the notable success achieved with the in‐clinic app, the study is limited by the relatively small sample size of only three physical therapists participating in the formal usability evaluation. Recognizing the critical need for diverse perspectives and experiences, it is imperative to underscore the importance of a more expansive and inclusive participation of a broader cohort of physical therapists in further usability evaluations. A more comprehensive sample size would yield valuable insights, guiding and informing further enhancements to the app's usability and overall effectiveness. Regarding the usability evaluations of the at‐home app, it was noted that a portion of the participants opted not to complete the usability questionnaires. They mentioned the questionnaire was very extensive. However, it is worth noting that despite this, the number of responses collected from the patients remained substantial.

## 5 Conclusion

This study focused on the development and usability of two different apps within an mHealth system designed to capture data from individuals with cLBP. One of the key findings was the high usability scores of both the in‐clinic and at‐home apps. The in‐clinic app was successfully utilized by physical therapists to conduct in‐clinic assessments. Similarly, the at‐home app enabled the cLBP patients to comply with filling out and submitting their EMA.

## Conflicts of Interest

The authors declare no conflicts of interest.
